# Clinical characteristics and risk factors of osteoporosis among older Asian men with type-2 diabetes mellitus, hypertension, or hyperlipidaemia

**DOI:** 10.1007/s11657-024-01442-y

**Published:** 2024-09-05

**Authors:** Yu Quan Tan, Ding Xuan Ng, Kalaipriya Gunasekaran, Weai Ling Lim, Ngiap Chuan Tan

**Affiliations:** 1 SingHealth Polyclinics, 167 Jalan Bukit Merah, Connection One (Tower 5), #15-10, Singapore, 150167 Singapore; 2 SingHealth Duke-NUS Family Medicine Academic Clinical Program, Singapore, Singapore

**Keywords:** Osteoporosis risk, Chronic diseases, Bone health, Fragility fractures, Singaporean men

## Abstract

***Summary*:**

This study investigated osteoporosis risk factors among older Asian men with type-2 diabetes mellitus, hypertension, or hyperlipidaemia in primary care. Advanced age, dementia, depression, and polypharmacy were associated with higher risks for osteoporosis. Screening strategies targeting these factors are crucial for improving bone health as part of comprehensive preventive care.

**Purpose:**

Asian patients with type-2 diabetes mellitus (T2DM), hypertension, or hyperlipidaemia (DHL) are predominantly managed in primary care. They are also at risk of osteoporosis, but men are often under-screened and under-treated for this preventable bone disorder. This study aimed to identify the clinical characteristics and risk factors of osteoporosis among older men with DHL in primary care for early intervention.

**Methods:**

This retrospective study included men aged 65 years and older managed in public primary care clinics for their DHL between 1st July 2017 and 30th June 2018. Demographic, clinical, laboratory, and imaging data were extracted from their electronic medical records based on their International Classification of Diseases-10 (ICD-10) diagnosis codes. Descriptive statistical analyses, with statistical significance set at *p* < 0.05, were conducted, followed by generalized estimating equation (GEE) modelling.

**Results:**

Medical records of 17,644 men (83.1% Chinese, 16.9% minority ethnic groups, median age 71 years) were analysed. 2.3% of them had diagnosis of osteoporosis, 0.15% had fragility fracture, and 26.0% of those diagnosed with osteoporosis were treated with bisphosphonates. Their mean HbA1c was 6.9%; mean systolic and diastolic blood pressure were 133 and 69 mmHg. The GEE model showed that age (OR = 1.07, 95%CI = 1.05–1.09, *p* < 0.001), dementia (OR = 2.24, 95%CI = 1.33–3.77, *p* = 0.002), depression (OR = 2.38, 95%CI = 1.03–5.50, *p* = 0.043), and polypharmacy (OR = 6.85, 95%CI = 3.07–15.26, *p* < 0.001) were significantly associated with higher risks for osteoporosis.

**Conclusion:**

Age, dementia, depression, and polypharmacy are associated with osteoporosis risks in men with DHL. Strategies to incorporate osteoporosis screening among older men with these risk factors are needed to improve their bone health.

**Supplementary Information:**

The online version contains supplementary material available at 10.1007/s11657-024-01442-y.

## Introduction

Osteoporosis, characterized by decreased bone density and increased susceptibility to fractures, poses a significant health hazard among the older population. While it has been extensively studied in postmenopausal women, there is a growing recognition of its impact on older men [1], particularly those with co-existing chronic diseases. Globally, the prevalence of osteoporosis among older men was reported to be 12.5% [2]. A recent study in 2020 projected a 23% increment in fragility fractures among 6 countries (France, Germany, Italy, Spain, the UK and Sweden) by 2030 [3]. Likewise in the USA, the number of hip fractures among men is expected to increase by 51.8% from 2010 to 2030 [4].

Men suffer from higher morbidity and mortality following fragility fractures, often experiencing greater functional disability and reliance on walking aids compared to women after suffering from vertebral fracture [5, 6]. Likewise, more men than women die at 1 year after a hip fracture, with a mortality rate of up to 37.5% in men [7]. Moreover, the risk of subsequent fractures following fragility fractures in males was found to be comparable to that of females [8]. In Singapore, the incidence of hip fractures has increased by 1.5 times in men and 5 times in women since the 1960s, a trend expected to escalate given the aging population of the country [9]. Consequently, osteoporosis in men is becoming a growing public health concern that requires attention.

Despite this, men are often overlooked in osteoporosis screening, diagnosis and management [5]. Studies have shown that a significant majority, ranging from 90 to 95%, of men did not receive a diagnosis or treatment for osteoporosis following fragility fractures [10, 11]. This oversight may stem from a lack of awareness about the condition among both physicians and patients, with physicians commonly associating osteoporosis with females and accorded low priority to assessing men’s bone health [12]. Men were also less cognizant of osteoporosis, its risk factors, and associated complications, thus diminishing their perceived need for screening [13]. Additionally, inadequate coordination among healthcare providers leads to a lack of continuity in osteoporosis management following fractures [14].

Early detection of osteoporosis in older men is essential for timely intervention to improve their bone health and prevent potential fractures. While some risk factors are non-modifiable, such as family history, prior fragility fractures, and aging, others like smoking, alcohol consumption, low BMI, sedentary lifestyle, and inadequate intake of calcium and vitamin D can be addressed by healthcare professionals. Furthermore, secondary causes including long-term corticosteroid or anticonvulsant use, and diseases like rheumatoid arthritis, diabetes, dementia, poor neuromuscular function such as Parkinson’s disease and stroke, renal disease, and hyperthyroidism can also contribute to increased risk [15, 16]. Some studies have also reported that depression is associated with a reduction in bone mineral density [17]. Diabetes is associated with an increased risk of osteoporosis and fragility fracture [18], with variable effects observed for different oral anti-glycaemic agents on osteoporosis and fragility fracture risk [19]. Hypertension and hyperlipidaemia are also implicated in osteoporosis risk, though the severity of hypertension and the association of specific lipid profiles with osteoporosis remain unclear [20–22].

As the burden of chronic illnesses grows, primary care physicians play a crucial role in managing and coordinating care for complex patients with multimorbidity [23]. There is a growing recognition of the association between osteoporosis and metabolic conditions such as diabetes, hypertension, and hyperlipidaemia, which are predominantly managed in primary care settings and are more prevalent in men [24]. Individuals with these conditions often exhibit systemic inflammation, oxidative stress, insulin resistance, and endothelial dysfunction, all of which can contribute to impaired bone re-modelling and decreased bone mineral density [21, 25, 26]. Understanding the interplay between osteoporosis and chronic diseases is crucial for developing effective preventive and management strategies tailored to the unique needs of elderly men. This underscores the importance of evaluating the clinical characteristics of this group of men with osteoporosis within the primary care setting. Primary care serves as the cornerstone of comprehensive healthcare delivery, offering ongoing management of chronic conditions and preventive care services. Integrating osteoporosis screening into routine primary care visits enables a holistic approach to patient care, addressing multiple health conditions simultaneously.

Therefore, the primary objective of the study is to identify the clinical characteristics and risk factors of osteoporosis among multi-ethnic older men with diabetes, hypertension, or hyperlipidaemia (DHL) in a primary care setting. This study also aims to explore the treatment rate of osteoporosis among this group of men. The secondary objective is to evaluate the association of control of DHL and types of chronic medications with the risk of osteoporosis. Insights from this study will allow primary care physicians to identify and prioritize the group of men requiring osteoporosis screening during chronic review and optimize their preventive health.

## Methods

### Study setting and population

This retrospective study included Singaporean and Singapore Permanent Resident men aged 65 years and above who had at least 2 visits to any of the 8 SingHealth Polyclinics (SHP) between 1st July 2017 and 30th June 2018.

Patients with osteoporosis were identified via 4 methods: with the diagnosis code of osteoporosis or osteoporotic fracture, on osteoporosis treatment medication, had a previous bone mineral density (BMD) test with a T score of less than − 2.5 or sustained a fragility fracture during the period of study [27]. Measures were taken to avoid double counting of patients identified with osteoporosis from either of the categories.

Fragility fracture was defined as a fracture of the vertebral, hip, femur, pelvis, humerus or wrist that occurred spontaneously or after a low energy trauma [27]. Identification of fragility fractures in this study was based on matching the clinical history documented on the x-ray order form with the corresponding radiological confirmation of fracture at the respective site.

Osteoporosis treatment medications were defined as bisphosphonates (alendronate, risedronate) and denosumab, which could be prescribed in SHP.

### Data sources

Data extracted from this study were from healthcare electronic platforms Sunrise Clinical Manager (SCM), Outpatient Administrative System (OAS) and Electronic Health Intelligence System (eHINTS). Visit diagnosis coding, diagnostic tests ordering, medications prescribing, documentation of medical notes and referrals were made by doctors via the SCM. Patients’ appointments and billing were managed by the OAS.

### Data collection

Clinical data of the study population was extracted from the databases and transformed to its desired, actionable format via a process known as the extract, transform, and load (ETL) database function. The diagnosis codes of osteoporosis and other co-morbidity conditions were extracted based on International Classification of Diseases-10 (ICD-10) diagnosis codes. Height, weight, BMD and x-rays reports, laboratory results, as well as electronic drug prescriptions were extracted from the SCM. Demographics such as age, ethnicity, and nationality were obtained from the OAS. Osteoporosis treatment medications were extracted up to 1 year after the study period. The transformed data was loaded into eHINTS and retrieved by the research team for further data analysis.

BMD results were uploaded into SCM as a free-text data field in a semi-structured report. The report included the indication of the scan and overall impression of the BMD results. Natural language processing (NLP) was employed to transform the BMD results in their free-text data reports into a quantifiable format on an Excel sheet for data analysis.

X-ray reports relating to images of regions around the spine (cervical, thoracic, lumbar), pelvis, hip, humerus and extremities (hand, wrist) were extracted. The identification of fragility fractures at the sites was confirmed by manual reviews of the x-ray reports by the principal investigator of the study.

HbA1c, lipids, and blood pressure readings were categorized according to controlled and uncontrolled based on existing local and institutional guidelines at the time of study period. Controlled diabetes was defined as HbA1c of ≤ 7% for < 75 years old, ≤ 7.5% for 75–79 years old, and ≤ 8% for 80 years and above [28]. Controlled hypertension was defined as < 130/80 mmHg with moderate to severe albuminuria (urine ACR > 3 mg/mmol; PCR > 15 mg/mmol), < 140/80 mmHg with diabetes, < 150/90 mmHg for 80 years and above, and < 140/90 mmHg for others [29]. Controlled LDL level was defined as < 4.1 mmol/L with hyperlipidaemia only, < 2.6 mmol/L with DM or stage 3 chronic kidney disease or worse and < 2.1 mmol/L with cardiovascular complications (ischaemic heart disease, previous stroke, peripheral vascular disease) or diabetes with stage 3 chronic kidney disease or worse. Controlled HDL level was defined as > 1.0 mmol/L. Controlled triglycerides level was defined as < 1.7 mmol/L [30]. Polypharmacy was defined as being on 5 or more medications [31].

### Data processing and audit

Research informatics staff from the research department in SHP ensured that the data extraction methodology was sound. An appointed trusted third party (TTP) from the SingHealth Cluster assisted to de-identify the data. The de-identified data was passed to the research team for analysis via secure file transfer protocol from the TPP.

### Statistical analyses

This study investigated the relationship between demographics and clinical characteristics of patients concerning osteoporosis. Descriptive statistics were employed for both categorical and continuous variables, with frequencies and percentages utilized for the former and median or mean for the latter, depending on the normality of data. Categorical data underwent Chi-squared or Fisher’s exact test, while continuous data underwent *t* test or Mann–Whitney *U* test as appropriate.

To enhance model interpretability and mitigate over-fitting, recursive feature elimination was implemented for feature selection in generalized estimating equation (GEE) modelling. Clinical inputs were carefully considered to ensure the inclusion of justified variables. The GEE model employed a correlation structure of independence, which accounted for the relationships between primary care clinics. Variance inflation factor was used to identify and address multicollinearity between variables. The estimated odds ratio, 95% confidence intervals and *p* values for each feature were reported, with statistical significance set at *p* < 0.05. All analysis were conducted using R version 4.2.3 and Python version 3.9.

### Ethical approval

This study was reviewed by the SingHealth Centralised Institutional Review Board (CIRB Ref: 2023/2046) for ethics review and deemed not require further ethical deliberation because of usage of de-identified data from a trusted third party.

## Results

### Demographic characteristics

**Fig. 1:**
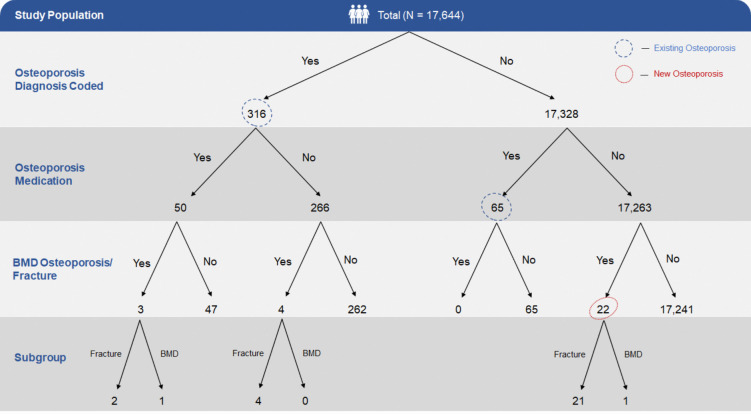
Breakdown of study population

A total of 17,644 men aged 65 and above with diabetes, hypertension, or hyperlipidaemia (DHL) were included for analysis (Fig. 1). A total of 403 of them were identified to have osteoporosis, of which 381 of them had an existing diagnosis of osteoporosis or on osteoporosis medications, and 22 of them were not diagnosed with osteoporosis after sustaining a fragility fracture or had BMD result showing osteoporosis (labelled as newly diagnosed osteoporosis). Table 1 summarized the demographics of the study population. Majority of them had hypertension (89%), followed by hyperlipidaemia (86.5%) and diabetes (41.9%). Patients were mostly Chinese (83.1%), Singapore citizen (97.9%), non-smoker (96.3%), and overweight (55.3%), with a median age of 71 years old (IQR = 68 to 77).

**Table 1:** Demographics of men with DHL aged 65 and above

	Overall
Total	17,644
Age (median [IQR])	71.00 [68.00, 77.00]
Ethnicity (%)	
Chinese	14,654 (83.1)
Malay	1511 (8.6)
Indian	916 (5.2)
Others	563 (3.1)
Nationality = Singaporean (%)	17,269 (97.9)
Smoking status (%)	
Non-smoker	14,638 (96.3)
Smoker	451 (3.0)
Ex-smoker (stopped > 6 months)	107 (0.7)
BMI (%)	
Underweight	733 (5.1)
Normal	4535 (31.4)
Overweight	7996 (55.3)
Obese	1187 (8.2)
Diabetes (%)	7387 (41.9)
Hypertension (%)	15,709 (89.0)
Hyperlipidaemia (%)	15,267 (86.5)
Systolic blood pressure, mmHg (mean [SD])	133 (17.0)
Diastolic blood pressure, mmHg (mean [SD])	69 (9.4)
HbA1c, % (mean [SD])	6.92 (1.23)

### Clinical characteristics associated with osteoporosis in men with DHL

The proportion of men with osteoporosis in the study population was 2.3% (Table 2). Factors significantly associated with osteoporosis at bivariate analysis included age, BMI, benign prostatic hyperplasia (BPH), dementia, depression, epilepsy, hypothyroid, Parkinson, stroke, chronic obstructive pulmonary disease (COPD), anti-depressant, proton pump inhibitor, sulfonylurea, metformin, HbA1c control, blood pressure control, and polypharmacy.

**Table 2:** Bivariate analysis of factors associated with osteoporosis in men with DHL (*N* = 17,644)

	Overall	No osteoporosis	Osteoporosis	*P* value
Total	17,644 (100)	17,241 (97.7)	403 (2.3)	
Age (median [IQR])	71.00 [68.00, 77.00]	71.00 [68.00, 77.00]	77.00 [70.00, 81.00]	< 0.001
Ethnicity (%)				0.066
Chinese	14,654 (83.1)	14,300 (82.9)	354 (87.8)	
Malay	1511 (8.6)	1489 (8.6)	22 (5.5)	
Indian	916 (5.2)	899 (5.2)	17 (4.2)	
Others	563 (3.2)	553 (3.2)	10 (2.5)	
Nationality = Singaporean (%)	17,269 (97.9)	16,874 (97.9)	395 (98.0)	0.982
Smoking status (%)				0.607
Non-smoker	14,638 (96.3)	14,293 (96.3)	345 (97.5)	
Ex-smoker (stopped > 6 months)	107 (0.7)	106 (0.7)	1 (0.3)	
Smoker	451 (3.0)	443 (3.0)	8 (2.3)	
BMI (%)				< 0.001
Underweight	733 (5.1)	701 (5.0)	32 (10.1)	
Normal	4535 (31.4)	4409 (31.2)	126 (39.6)	
Overweight	7996 (55.3)	7860 (55.6)	136 (42.8)	
Obese	1187 (8.2)	1163 (8.2)	24 (7.5)	
Comorbidities				
Anxiety = yes (%)	110 (0.6)	107 (0.6)	3 (0.7)	0.742
Asthma = yes (%)	705 (4.0)	683 (4.0)	22 (5.5)	0.165
BPH = yes (%)	1437 (8.1)	1389 (8.1)	48 (11.9)	0.007
IHD = yes (%)	4601 (26.1)	4479 (26.0)	122 (30.3)	0.060
Dementia = yes (%)	426 (2.4)	401 (2.3)	25 (6.2)	< 0.001
Depression = yes (%)	116 (0.7)	106 (0.6)	10 (2.5)	< 0.001
Epilepsy = yes (%)	94 (0.5)	88 (0.5)	6 (1.5)	0.020
Hypothyroid = yes (%)	477 (2.7)	459 (2.7)	18 (4.5)	0.040
Pre-diabetes = yes (%)	1984 (11.2)	1938 (11.2)	46 (11.4)	0.977
Osteoarthritis = yes (%)	3503 (19.9)	3409 (19.8)	94 (23.3)	0.088
Parkinson = yes (%)	290 (1.6)	277 (1.6)	13 (3.2)	0.020
Psoriasis = yes (%)	123 (0.7)	119 (0.7)	4 (1.0)	0.367
Schizophrenia = yes (%)	49 (0.3)	47 (0.3)	2 (0.5)	0.309
Rheumatoid arthritis = yes (%)	22 (0.1)	21 (0.1)	1 (0.2)	0.399
Stroke = yes (%)	2089 (11.8)	2016 (11.7)	73 (18.1)	< 0.001
Hyperthyroid = yes (%)	104 (0.6)	103 (0.6)	1 (0.2)	0.735
Gout = yes (%)	2076 (11.8)	2035 (11.8)	41 (10.2)	0.355
Cancer = yes (%)	946 (5.4)	921 (5.3)	25 (6.2)	0.518
PVD = yes (%)	404 (2.3)	389 (2.3)	15 (3.7)	0.076
TIA = yes (%)	475 (2.7)	466 (2.7)	9 (2.2)	0.674
Chronic liver disease = yes (%)	56 (0.3)	53 (0.3)	3 (0.7)	0.136
Anaemia = yes (%)	469 (2.7)	459 (2.7)	10 (2.5)	0.947
COPD = yes (%)	325 (1.8)	308 (1.8)	17 (4.2)	0.001
CKD (%)				0.437
Stage 1	1146 (6.5)	1123 (6.5)	23 (5.7)	
Stage 2	6001 (34.0)	5858 (34.0)	143 (35.5)	
Stage 3 and above	4148 (23.5)	4065 (23.5)	83 (20.5)	
Unknown	6349 (36.0)	6195 (35.9)	154 (38.2)	
Medications				
Anti-psychotic = yes (%)	29 (0.2)	27 (0.2)	2 (0.5)	0.141
Anti-depressant = yes (%)	114 (0.6)	106 (0.6)	8 (2.0)	0.002
Anti-convulsant = yes (%)	50 (0.3)	47 (0.3)	3 (0.7)	0.106
5 alpha reductase inhibitor = yes (%)	92 (0.5)	90 (0.5)	2 (0.5)	> 0.999
Proton pump inhibitor = yes (%)	3970 (22.5)	3835 (22.2)	135 (33.5)	< 0.001
Sulfonylurea = yes (%)	2833 (16.1)	2797 (16.2)	36 (8.9)	< 0.001
Dipeptidyl peptidase-4 inhibitor = yes (%)	761 (4.3)	751 (4.4)	10 (2.5)	0.088
Sodium glucose-2 inhibitor = yes (%)	27 (0.2)	27 (0.2)	0 (0.0)	> 0.999
Insulin = yes (%)	629 (3.6)	622 (3.6)	7 (1.7)	0.062
Metformin = yes (%)	4050 (23.0)	3983 (23.1)	67 (16.6)	0.003
Prednisolone = yes (%)	332 (1.9)	322 (1.9)	10 (2.5)	0.477
Polypharmacy = yes (%)	150 (0.9)	139 (0.8)	11 (2.7)	< 0.001
Laboratory				
Urine protein/creatinine ratio = normal (%)	993 (45.2)	973 (45.2)	20 (43.5)	0.933
Urine albumin/creatinine ratio = normal (%)	4290 (50.5)	4212 (50.7)	78 (43.3)	0.061
Annual prednisolone dosage (mean (SD))	1.41 (12.52)	1.41 (12.51)	1.50 (12.84)	0.881
TSH status (%)				0.268
High	189 (43.2)	179 (42.4)	10 (62.5)	
Normal	191 (43.6)	186 (44.1)	5 (31.2)	
Low	58 (13.2)	57 (13.5)	1 (6.2)	
HbA1c = uncontrolled (%)	2055 (33.3)	2030 (33.6)	25 (20.3)	0.003
LDL = uncontrolled (%)	3377 (22.9)	3301 (22.9)	76 (22.7)	0.981
HDL = uncontrolled (%)	2242 (15.2)	2204 (15.3)	38 (11.3)	0.057
BP status = uncontrolled (%)	5593 (37.0)	5495 (37.2)	98 (27.9)	< 0.001
Triglycerides (%)				0.821
Controlled (< 1.7)	11,704 (79.3)	11,436 (79.2)	268 (80.0)	
Uncontrolled (1.7–4.5)	2985 (20.2)	2919 (20.2)	66 (19.7)	
Very uncontrolled (> 4.5)	77 (0.5)	76 (0.5)	1 (0.3)	

### GEE modelling on osteoporosis in men with DHL

The GEE modelling conducted showed that age (OR = 1.07, 95%CI = 1.05–1.09, *p* < 0.001), dementia (OR = 2.24, 95%CI = 1.33–3.77, *p* = 0.002), depression (OR = 2.38, 95%CI = 1.03–5.50, *p* = 0.043), proton pump inhibitor (OR = 1.52, 95%CI = 1.19–1.94, *p* = 0.001), and polypharmacy (OR = 6.85, 95%CI = 3.07–15.26, *p* < 0.001) remained significantly associated with higher odds for osteoporosis (Table 3). Patients who were overweight, with chronic kidney disease stage 3 and above, and on sulfonylurea were 30% (OR = 0.70, 95%CI = 0.54–0.90, *p* = 0.006), 45% (OR = 0.55, 95%CI = 0.31–0.99), *p* = 0.048), and 48% (OR = 0.52, 95%CI = 0.33–0.81, *p* = 0.005) less likely to have an osteoporosis diagnosis respectively. Blood pressure and HDL control status were not significantly associated with osteoporosis. An independence correlation structure between clusters yielded an estimated variation of 0.971 with a standard error of 0.72.

**Table 3:** GEE model on factors associated with osteoporosis in men with DHL

Variable	Odds ratio (95%CI)	*P* value
Age	1.067 (1.047–1.086)	< 0.001
BMI		
Underweight	1.413 (0.933–2.14)	0.102
Normal		
Overweight	0.696 (0.539–0.9)	0.006
Obese	0.893 (0.549–1.452)	0.648
Comorbidities		
BPH	1.125 (0.751–1.684)	0.568
Dementia	2.243 (1.333–3.773)	0.002
Depression	2.378 (1.028–5.497)	0.043
Stroke	1.296 (0.904–1.859)	0.158
COPD	1.492 (0.818–2.724)	0.192
Chronic kidney disease		
Stage 1	Ref	-
Stage 2	0.946 (0.554–1.614)	0.839
Stage 3 and above	0.553 (0.308–0.994)	0.048
Unknown	0.897 (0.523–1.538)	0.693
Medications		
Proton pump inhibitor	1.522 (1.191–1.944)	0.001
Sulfonylurea	0.521 (0.33–0.824)	0.005
Polypharmacy	6.845 (3.071–15.257)	< 0.001
Laboratory		
BP control		
Good	Ref	-
Poor	0.8 (0.599–1.068)	0.130
HDL control		
Good	Ref	-
Poor	0.893 (0.61–1.307)	0.560

### GEE modelling on diabetic men with osteoporosis

To examine the robustness of the findings, sensitivity analysis was conducted on diabetic patients with a similar correlation structure involving the addition of the HbA1c control status of diabetic patients into the model (Table 4). Results from the analysis showed that the 4 variables of age, dementia, depression, and polypharmacy remained significant, with increased odds of osteoporosis. Blood pressure, HDL level, and HbA1c control were not significantly associated with osteoporosis. This model also shows that correlation estimated variance remains similar at 0.978, with a standard error of 1.54.

**Table 4:** GEE model on factors associated with osteoporosis for men with diabetes

Variable	Odds ratio (95%CI)	*P* value
Age	1.063 (1.031–1.097)	< 0.001
BMI		
Underweight	1.507 (0.634–3.581)	0.353
Normal	Ref	-
Overweight	0.679 (0.416–1.109)	0.122
Obese	0.858 (0.405–1.817)	0.69
Comorbidities		
BPH	1.1 (0.543–2.226)	0.792
Dementia	2.865 (1.395–5.886)	0.004
Depression	3.619 (1.047–12.504)	0.042
Stroke	1.243 (0.687–2.251)	0.472
COPD	1.562 (0.462–5.283)	0.473
Chronic kidney disease		
Stage 1	Ref	-
Stage 2	1.095 (0.465–2.583)	0.835
Stage 3 and above	0.437 (0.165–1.159)	0.096
Unknown	0.656 (0.243–1.771)	0.405
Medications		
Proton pump inhibitor	1.247 (0.815–1.906)	0.309
Sulfonylurea	0.631 (0.373–1.067)	0.086
Polypharmacy	10.515 (4.18–26.452)	< 0.001
Laboratory		
BP control		
Good	Ref	-
Poor	1.003 (0.632–1.594)	0.989
HDL control		
Good	Ref	-
Poor	1.059 (0.606–1.853)	0.840
HbA1c control		
Good	Ref	-
Poor	0.623 (0.359–1.084)	0.094

### Prescribed medications and supplements for men with osteoporosis

This study compared the osteoporosis supplements prescribed to the new (*n* = 22) and existing (*n* = 381) men with osteoporosis. The results showed that a lower proportion of those newly diagnosed patients (31.8%) were on medication or supplements compared to the existing patients (47.2%). Calcium carbonate/vitamin D tablet was the most prescribed supplement in both groups. The treatment rate for existing osteoporosis was 26%, with 82.8% of them prescribed alendronate and 17.2% of them were prescribed risedronate. Twenty-two out of 403 (5.5%) were not diagnosed with osteoporosis and were not started on osteoporosis treatment despite having BMD results showing osteoporosis or a history of fragility fracture (Supplementary table S1 and S2).

### Demographic of men with newly diagnosed osteoporosis with fracture

Further breakdown of men with newly diagnosed osteoporosis who sustained a fragility fracture during study period revealed that they were mostly Chinese (85.7%), overweight (64.7%), and non-smokers (88.9%), with a median age of 76 years old (Table 5).

**Table 5:** Demographic of men with newly diagnosed osteoporosis with fragility fracture (*N* = 21)

Variable	Overall
Total	21 (100)
Age (median [IQR])	76.00 [70.00, 79.00]
Ethnicity (%)	
Chinese	18 (85.7)
Indian	1 (4.8)
Others	2 (9.5)
Nationality = Singaporean (%)	20 (95.2)
BMI (%)	
Normal	4 (23.5)
Overweight	11 (64.7)
Obese	2 (11.8)
Smoking status (%)	
Ex-smoker	1 (5.6)
Non-smoker	16 (88.9)
Smoker	1 (5.6)

## Discussion

In this retrospective study, clinical characteristics, risk factors, and treatment rate for osteoporosis were analyzed among men aged 65 years and above with DHL. Consistent with previous research, several significant factors like older age, dementia, depression, and polypharmacy are found to be associated with osteoporosis in older men. The association between depression and osteoporosis in men has been explored in numerous studies, with conflicting findings reported. While some studies have found no significant correlation, others have suggested potential association, particularly younger men [32–34]. Depression can lead to hormonal changes such as an increase in cortisol and a decrease in sex hormones, causing reduced in bone mineral density and increased osteoporosis risk [17]. This is in line with this study which reported depression to have a higher odd of 3.6 for osteoporosis among the older group of men above 65 years old in the study population, suggesting a potential correlation of depression with osteoporosis in older men.

Osteoporosis and dementia are common geriatric syndromes. Studies have shown a bidirectional relationship between these two conditions. Both conditions share similar risk factors such as advanced age, vitamin D deficiency, physical inactivity, polypharmacy, and falls risk. However, this correlation was not previously well-studied in older men [35, 36]. This study reported an increased odds of osteoporosis among older men with dementia, suggesting that the correlation may not be gender specific to women. Polypharmacy emerged as another significant risk factor for osteoporosis, aligning with previous literature relating polypharmacy to an increased risk of falls and subsequent fractures [37]. This emphasizes the importance of comprehensive assessment and management strategies addressing such risk factors in older men. The GEE model had high correlation estimated variance suggesting high variation in the outcome of osteoporosis between clinics. This could be due to different patient demographics and osteoporosis screening practices between clinics.

The current study shed light on the characteristics of older men who sustained fragility fracture during the study period. Contrary to the established risk factors like smoking and low BMI [38], it was observed that the individuals who sustained fragility fractures were predominantly overweight and non-smoker. This challenges conventional understanding and prompts further exploration into the complex interplay of fracture risk factors. A possible explanation of such disparity is the underestimation of smoking prevalence within the study population, despite national data indicating 16% of the male population in Singapore were reported to be daily smoker, with 33.8% of them within the age group of 60 years old and above [39]. Moreover, the study focused on individuals with DHL, with a substantial proportion (63.5%) being overweight or obese at baseline. This demographic profile may have influenced the observed association between fragility fractures and overweight status. However, emerging evidence suggests an increased risk of fractures in obese individuals, attributed to underlying mechanisms such as chronic proinflammatory status, insulin resistance, obesity-related reduction in testosterone levels in men, and altered adipokine secretion [40].

This study had several strengths. This is one of the pioneering studies focusing on clinical characteristics of osteoporosis among older men with DHL in Singapore, contributing to the limited evidence. The strengths of this study include a large retrospective cohort study with a considerable sample size. This study supported the under-diagnosis and under-treatment of osteoporosis in men, with only 26% of older men diagnosed with osteoporosis received related treatment. Notably, among those newly diagnosed during the study period, none received treatment within a year post-diagnosis, underscoring a significant treatment gap for osteoporosis in this cohort. The treatment rate for newly diagnosed cases might be underestimated, particularly as many were diagnosed after experiencing fragility fractures, leading them to seek acute management in emergency departments and subsequent orthopaedic specialist care.

However, there were also limitations to this study. The reliance on available data from healthcare databases limited the inclusion of crucial risk factors for osteoporosis such as exercise, diet, and alcohol history, potentially affecting the comprehensiveness of the analysis. Despite the association of osteoporosis with diabetes, hypertension, and hyperlipidaemia, the proportion of osteoporosis among men with such comorbidities was 2.3% in this study. Globally, the prevalence of osteoporosis among older men with diabetes was reported 23% [41]. Locally, the prevalence of femoral neck and spine osteoporosis was reported to be 8.5% and 2.9% respectively among healthy men with no chronic medical conditions [42]. The low prevalence could be attributed to the paucity of screening and osteoporosis risk review of these men, whose attending primary care physicians could have been pre-occupied with managing their other co-morbidities. This study revealed similar risk factors for osteoporosis between men with DHL and men in general. However, with the baseline increased risk of osteoporosis due to DHL, it is important for case finding among men with such co-morbidity when they visit their primary care physicians for routine follow up of their chronic conditions to enhance their preventive screening.

Patients with osteoporosis were identified using four methods including diagnosis code, BMD results, history of fragility fracture, and anti-osteoporotic medication, ensuring thorough screening and identification of the study population. However, despite the best efforts to identify cases with osteoporosis, there may be underlying biases as some patients labelled without osteoporosis may have underlying osteoporosis due to the limitations from a retrospective study. The findings from this study may not be generalisable but could provide a valuable foundation for future prospective studies in this area.

The study population data was extracted from 1st July 2017 to 30th June 2018, predating the publication of a new guideline on osteoporosis identification and management in primary care in Singapore in November 2018 [43]. A recent local study reported that many primary care physicians reported good knowledge and utilization of local osteoporosis guidelines [44]. Nonetheless, awareness of these guidelines does not always translate into better clinical practice due to multiple factors. They continue to face challenges, such as limited consultation time and frequent distraction or prioritization of management of metabolic diseases over osteoporosis risk review. A significant revamp of the primary healthcare eco-system to focus on preventive health via multidisciplinary care teams is needed to create an impactful paradigm shift to optimize osteoporosis management.

## Conclusion

This study identified the association of age, depression, dementia, and polypharmacy with osteoporosis in men with diabetes, hypertension, and hyperlipidaemia. The findings underscore the importance of opportunistic case finding for osteoporosis among elderly men with such co-morbidity to increase screening rate. Seamless integration of tailored screening protocols into routine care practices is recommended to enable early identification of osteoporosis and facilitate timely interventions aimed at preventing fragility fractures and improving bone health in this vulnerable population. Future research should consider prospective studies to validate an Asian male self-osteoporosis risk calculator encompassing clinical, lifestyle factors for its earlier identification in men.

## Supplementary Information

Below is the link to the electronic supplementary material.

Supplementary file1 (DOCX 16 KB)

## Data Availability

The dataset analyzed during the study are not publicly available due to privacy and confidentiality concerns but are available from the corresponding author on reasonable request upon review.
