# Supplementary material for: Clinical characteristics and risk factors of osteoporosis among older Asian men with type-2 diabetes mellitus, hypertension, or hyperlipidaemia
Source: Arch Osteoporos. 2024 Sep 5;19(1):83. doi: 10.1007/s11657-024-01442-y (PMC11377474; doi:10.1007/s11657-024-01442-y)
Supplement: Supplementary file 1 — Supplementary file1 (DOCX 16 KB) [file 11657_2024_1442_MOESM1_ESM.docx]

**Supplementary tables**

| **Table S1: Osteoporosis medications and supplements for men with newly diagnosed osteoporosis (N = 22)** | | |
| --- | --- | --- |
| **Variable** | **Frequency** | **Percentage (%)** |
| **Total** | 22 | 100 |
| **Osteoporosis related medication and supplement** | |  |
| No | 15 | 68.2 |
| Yes | 7 | 31.8 |
| **Number of Osteoporosis related medication and supplement** | | |
| 0 | 15 | 68.2 |
| 1 | 6 | 27.3 |
| 2 or more | 1 | 4.5 |
|  |  |  |
| **Medications and Supplements** |  |  |
| **Calcium Carb 450mg, Vit D 200 unit Tab** |  |  |
| No | 16 | 72.7 |
| Yes | 6 | 27.3 |
| **Calcium Carbonate Tablet** |  |  |
| No | 21 | 95.5 |
| Yes | 1 | 4.5 |
| **Colecalciferol [Vitamin D3] Capsule/Tab** |  |  |
| No | 21 | 95.5 |
| Yes | 1 | 4.5 |

| **Table S2: Osteoporosis medications and supplements for men with existing osteoporosis (N = 381)** | | |
| --- | --- | --- |
| **Variable** | **Frequency** | **Percentage (%)** |
| **Total** | 381 | 100 |
| **Osteoporosis related medication and supplement** |  |  |
| No | 201 | 52.8 |
| Yes | 180 | 47.2 |
| **Number of Osteoporosis related medication and supplement** |  |  |
| 0 | 201 | 52.8 |
| 1 | 86 | 22.6 |
| 2 or more | 94 | 24.7 |
|  |  |  |
| **Medications and Supplements** |  |  |
| **Calcium Carb 450mg, Vit D 200 unit Tab** |  |  |
| No | 231 | 60.6 |
| Yes | 150 | 39.4 |
| **Colecalciferol [Vitamin D3] Capsule/Tab** |  |  |
| No | 342 | 89.8 |
| Yes | 39 | 10.2 |
| **Risedronate Sodium Tablet** |  |  |
| No | 364 | 95.5 |
| Yes | 17 | 4.5 |
| **Alendronic Acid Tablet** |  |  |
| No | 299 | 78.5 |
| Yes | 82 | 21.5 |
| **Calcium Carbonate Tablet** |  |  |
| No | 368 | 96.6 |
| Yes | 13 | 3.4 |
